# Intracellular and Extracellular Vesicle miRNA Signatures in Human iPSC‐Derived Neural Stem Cells and Floor Plate Progenitors

**DOI:** 10.1096/fj.202501157R

**Published:** 2025-08-28

**Authors:** Lilian Cruz, Frederico Moraes Ferreira, Camila Miranda Lopes‐Ramos, Zhiyun Wei, William T. Hendriks, H. A. Jinnah, Helder I. Nakaya, D. Cristopher Bragg, Anna M. Krichevsky, Xandra Owens Breakefield, Marilene Hohmuth Lopes

**Affiliations:** ^1^ Department of Cell and Developmental Biology Institute of Biomedical Sciences, University of São Paulo São Paulo Brazil; ^2^ Department of Neurology, Massachusetts General Hospital and Program in Neuroscience Harvard Medical School Charlestown Massachusetts USA; ^3^ Cellular, Genetic, and Molecular Nephrology Laboratory (LIM‐29), Hospital das Clínicas University of São Paulo Medical School São Paulo Brazil; ^4^ Institute for Investigation in Immunology, INCT São Paulo Brazil; ^5^ Department of Biostatistics Harvard T.H. Chan School of Public Health Boston Massachusetts USA; ^6^ Channing Division of Network Medicine, Department of Medicine Brigham and Women's Hospital and Harvard Medical School Boston Massachusetts USA; ^7^ Department of Neurology Brigham and Women's Hospital and Harvard Medical School, HMS Initiative for RNA Medicine Boston Massachusetts USA; ^8^ Department of Neurology Emory University Atlanta Georgia USA; ^9^ Department of Human Genetics Emory University Atlanta Georgia USA; ^10^ Department of Pediatrics Emory University Atlanta Georgia USA; ^11^ Department of Clinical and Toxicological Analyses School of Pharmaceutical Sciences, University of São Paulo São Paulo Brazil; ^12^ Hospital Israelita Albert Einstein São Paulo SP Brazil; ^13^ Department of Radiology Massachusetts General Hospital, Harvard Medical School Charlestown Massachusetts USA

**Keywords:** dopaminergic neurons, EXOC5, exosomes, floor plate progenitors, human induced pluripotent stem cells, microRNA, neural differentiation, neuronal development

## Abstract

Refined control of intrinsic and extrinsic signals is critical for specific neuronal differentiation. Here, we differentiated human induced pluripotent stem cells (hiPSCs) from three different healthy donors into neural stem cells (NSCs) and floor plate progenitors (FPPs; progenitors of dopaminergic neurons) and further performed intracellular and extracellular vesicles' (EVs) miRNA profiling. While NSC and FPP cells differed significantly in levels of only 8 intracellular miRNAs, their differences were more evident in the EV miRNAs with 27 differentially expressed miRNAs. Target validation of intracellular miRNAs revealed that FPPs expressed more *EXOC5* mRNA than NSCs, which is implicated in the function of primary cilia, an essential signaling organelle in FPPs. Moreover, we found a group of 5 miRNAs consistently enriched in EVs from these three cell types. This study presents a foundation for the field of miRNA regulation in neural development and provides new insights for disease modeling and regenerative medicine.

## Graphical abstract

Human induced pluripotent stem cells were differentiated into neural stem cells and floor plate progenitors of dopaminergic neurons, followed by microRNA profiling. While intracellular differences were limited, extracellular vesicle microRNAs showed 27 differentially expressed microRNAs and five consistently enriched across lineages. Target analysis revealed upregulation of Exocyst Complex Component 5 in progenitors, linked to primary cilia, offering insight into neural fate regulation and selective vulnerability of dopaminergic neurons in Parkinson's disease.

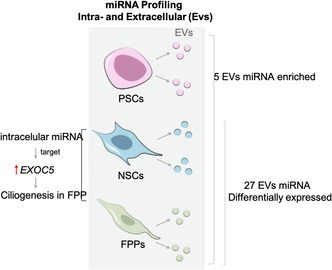

## 1 Introduction

The complexity of the human nervous system is reflected by its wide variety of neuronal types. They are defined early in development when neural stem cells, with the potential to differentiate into a broad spectrum of neural cell types (neurons, astrocytes, and oligodendrocytes), commit to a specific neuronal phenotype. These early embryonic events are consequences of regionally specific, morphogen‐regulated transcriptional networks established in a time‐dependent and spatially controlled environment [1, 2, 3]. In the developing embryo, a group of cells located at the ventral midline of the neural tube, the floor plate, acts as a source of ventral midbrain dopaminergic neurons, the neurons affected in the neurodegenerative disorder, Parkinson's Disease (PD) [4]. These specialized sets of cells not only have the neurogenic potential but are considered an organizing center for brain development. They govern the specification of neuronal and glial identities through the secretion of the glycoprotein sonic hedgehog (SHH) and direct axonal trajectories through the secretion of SHH and netrin 1 [5, 6].

Previous studies have demonstrated that regulatory non‐coding RNAs, including micro RNAs (miRNAs), are dynamically regulated during neural development, indicating that miRNAs have a role in the switch of transcriptional programs during the process of stem cell differentiation. External signals elicit the expression of different miRNA sets, which act synergistically with other transcriptional regulators, such as transcription and epigenetic factors, to establish regulatory networks in the specification of neuronal subtypes [7, 8, 9]. miRNAs circulate in a highly stable cell‐free manner in biological fluids and can be significantly altered in a wide diversity of physiological and pathological conditions [10, 11]. One possible secretion mechanism of both morphogens and miRNAs is through extracellular vesicles (EVs), such as exosomes and microvesicles, suggesting that these active molecules can be delivered to target cells [12, 13]. In the recipient cell, the EV‐delivered functional cargo can regulate gene expression, induce signaling pathways, and consequently modulate physiological processes [14, 15, 16].

As stem cell fate is continuously adjusted by specific conditions of the microenvironment within which they reside, miRNAs emerge as fundamental intrinsic and extrinsic developmental regulatory clues. Furthermore, due to their specialized organizing functions and their spatial location in the developing neural tube, floor plate cells possess remarkable secretory and signaling properties. Although very few studies suggest that other extracellular factors besides morphogens can contribute to neural tube formation and patterning [17, 18], the role of EVs and secreted regulatory RNA in this process has not yet been fully addressed. Thus, uncovering additional extrinsic signaling molecules released by floor plate progenitors becomes a critical topic in developmental biology and may provide a foundation for novel therapies in diseases affecting dopaminergic neurons, such as PD [4].

Here, we used human induced pluripotent stem cells (hiPSCs) from three different healthy subjects as an in vitro platform to model the early stages of human development. We differentiated the hiPSCs into two neural cell types: neural stem cells (NSCs), which are not committed to a specific neural cell type, and floor plate progenitors (FPPs), which are committed to a dopaminergic neuronal phenotype. We next screened intracellular and secreted EV miRNAs in these three cell populations (hiPSCs, NSCs, and FPPs) to determine potential signatures among these neuronal stages. The present findings are useful not only as a resource but also provide new insights to improve differentiation protocols, establish disease models in vitro, and further explore novel drugs and cell therapies for neurological diseases.

## 2 Methods

### 2.1 hiPSCs Culture

hiPSCs were generated by reprogramming skin fibroblasts of male individuals through an miRNA transfection method [19, 20]. Experiments were performed with cells derived from three healthy subjects (biological replicates) (C1.03, C2.04, C3.05). hiPSC lines were cultured in the E8 medium consisting of Essential 8 Basal Medium with Essential 8 Supplement and penicillin/streptomycin (1%) (Life Technologies) on Geltrex (Life Technologies)‐coated tissue culture plates. hiPSCs were sub‐cultured weekly as cell clumps by PBS‐EDTA 0.5 mM dissociation, followed by scraping off hiPSC colonies and transferring them to new Geltrex‐coated plates at a 1:5–1:10 ratio. Cell lines were confirmed to have a normal karyotype by Cell Line Genetics Inc. (Madison, WI, USA). All procedures involving human cells were approved by the institutional ethics committee, as previously described [20].

### 2.2 hiPSC Differentiation Into NSCs

hiPSCs were seeded as colonies on Geltrex‐coated plates in the E8 Medium. At a confluency of 10%–20%, the E8 medium was switched to the Gibco PSC Neural Induction Medium (Life Technologies) containing the Neurobasal medium and Gibco PSC Neural Induction Supplement for seven days with medium exchanges every other day. On day 7, cells were dissociated using Accutase (Life Technologies) and seeded onto Geltrex‐coated plates in the neural expansion medium consisting of the Neurobasal Medium: Advanced DMEM/F12 (1:1) supplemented with 2% PSC Neural Induction Supplement and 5 μM ROCK inhibitor Y‐27632 (Sigma‐Aldrich, St. Louis, MO). After 24 h, the medium was exchanged to remove the ROCK inhibitor. The neural expansion medium was changed every other day, and NSCs were sub‐cultured every 4–5 days [21]. NSCs were maintained on Geltrex‐coated plates in the neural expansion medium for up to 4 or 5 passages.

### 2.3 hiPSC Differentiation Into FPPs and Dopaminergic Neurons

hiPSC differentiation into FPPs and dopaminergic neurons was performed according to the instructions of the PSC Dopaminergic Neuron Differentiation Kit (Life Technologies). Briefly, hiPSCs were seeded as single cells on vitronectin‐coated plates in the E8 medium with the 10 μM ROCK inhibitor Y‐27632. After 24 h, the medium was replaced with the Floor Plate Specification Medium (Neurobasal Medium with 20X Floor Plate Specification Supplement) and exchanged on days 3, 5, 7, and 9 (Specification step). On day 10, cells were dissociated with Accutase and plated on laminin‐coated plates using the Floor Plate Expansion Medium (Floor Plate Cell Expansion Base Medium with 50X Floor Plate Cell Expansion Supplement) with medium exchange every other day until confluency after 4–5 days (Expansion step). FPPs were maintained on laminin‐coated plates in the Floor Plate Expansion Medium for up to 4 or 5 passages. For differentiation into dopaminergic neurons (tyrosine hydroxylase‐positive neurons), adherent FPPs were dissociated with Accutase and cultured in suspension to form neurospheres in low‐attachment plastic culture plates. The Floor Plate Expansion Medium was exchanged every other day, and after 5 days, neurospheres were dissociated with Accutase and re‐seeded on Poly‐D‐lysine/Laminin‐coated plates using the Dopaminergic Neuron Maturation Medium (DMEM/F‐12 medium with 50X Dopaminergic Neuron Maturation Supplement). Half‐volume medium change was performed every 2–3 days for 14 days (Maturation step). Cell identity at each stage was confirmed by immunofluorescence using well‐established markers: Nanog, Sox2, Oct4, and TRA‐1‐60 for hiPSCs; Sox1, Nestin, Pax6, and Msi1 for NSCs; and Lmx1a, Foxa2, and Otx2 for FPPs. These profiles, along with consistent differentiation outcomes across lines and further validation at later stages (e.g., TH+ dopaminergic neurons), support the specificity and robustness of the differentiation protocol.

### 2.4 Immunofluorescence

Immunostaining was performed to confirm expression of pluripotent markers in hiPSC lines, neural markers in NSCs, midbrain‐specified FPP markers in FPPs, and dopaminergic neuronal markers in fully differentiated dopaminergic neurons. Briefly, cells were seeded on coverslips pre‐treated with Geltrex (hiPSCs and NSCs) or laminin (FPPs) in their respective media for 48 h. Cells were fixed with 4% paraformaldehyde for 20 min at room temperature. After three washes with PBS buffer (Invitrogen, Carlsbad, CA, USA), the cells were blocked and permeabilized with PBS containing 0.3% Triton X‐100 and 5% BSA for 1 h at room temperature. The blocking buffer was removed, and cells were incubated in primary antibodies diluted in PBS containing 0.1% Triton X‐100 and 1% BSA at the specified dilution overnight at 4°C. Coverslips were washed 3× in PBS, incubated in secondary antibodies for 1 h at room temperature, and nuclei were counterstained by using 4′,6‐Diamidino‐2‐Phenylindole, Dilactate (DAPI—1:5000, Thermo Scientific, D3571). Coverslips were mounted on slides using the VECTASHIELD Antifade Mounting Medium (Vector Laboratories, Burlingame, CA, H1000‐10). Primary antibodies and dilutions consisted of rabbit anti‐Nanog (Abcam ab21624; 1:100), rabbit anti‐Oct3/4 (Abcam, ab19857; 1:100), mouse anti‐Sox2 (1:200), mouse anti‐Tra‐1‐60 (EMD Millipore MAB4360; 1:200), mouse anti‐Nestin (Abcam, ab21628; 1:200), rabbit anti‐Musashi (Abcam, ab21628; 1:100), rabbit anti‐Sox1 (Abcam, ab87775; 1:250), rabbit anti‐Pax6 (1:200), mouse anti‐βIII Tubulin (1:200), mouse anti‐Foxa2 (1:2000), goat anti‐Otx2 (1:250), rabbit anti‐Lmx1a (1:100), mouse anti‐MAP2 (1:200), rabbit anti‐TH (1:1000) (Human Dopaminergic Neuron Immunocytochemistry Kit, A29515), and rabbit anti‐acetyl‐alpha‐tubulin (Cell Signaling, 5335T; 1:500). Secondary antibodies were goat anti‐rabbit Alexa Fluor 488 (A‐11008), goat anti‐rabbit Alexa Fluor 594 (A‐11012), goat anti‐rat Alexa Fluor 594 (A‐11007), goat anti‐mouse Alexa Fluor 488 (A11001), goat anti‐mouse Alexa Fluor 594 (A‐11032), and donkey anti‐rabbit Alexa Fluor 488 (A‐21206). Secondary antibodies were all from Thermo Scientific and used at a final dilution of 1:1000. Images were acquired using either an epifluorescence microscope (Zeiss Axio Imager M2) using 10× or 20× objective or a Zeiss LSM 800 Airyscan confocal microscope (Carl Zeiss Microscopy GmbH, Jena, Germany) using 63× objective. Scale bars were included in all figures as indicated in each legend. Images were minimally processed for brightness and contrast using FIJI (ImageJ), and all adjustments were applied equally to the entire images.

### 2.5 Conditioned Media Collection and EV Isolation

Conditioned media (CM) from hiPSCs, NSCs, and FPPs cultured in the E8, Neural Expansion Medium, and Floor Plate Expansion Medium, respectively, were harvested and processed immediately through sequential centrifugation steps prior to storage in a −80°C freezer. All cell culture conditions and media composition were serum‐free. CM were centrifuged at 300 *g* for 10 min at 4°C, and then the supernatants were collected and centrifuged at 2000 *g* for 20 min at 4°C to remove contaminating cellular fragments and cell debris. The supernatants were collected and subsequently filtered through a 0.8 μm pore size filter (Merck Millipore, Bayswater, Victoria, Australia). The clarified CM was stored at −80°C until EV isolation. Prior to isolation, CM were thawed in a water bath at room temperature, and EVs were purified from 32 mL of CM using the exoEasy Maxi Kit (Qiagen, Hilden, Germany) according to the manufacturer's instructions with a few modifications in the final step. Basically, instead of eluting the EVs with the XE Elution Buffer from the kit, 400 μL of Lysis Buffer from the mirCURY RNA isolation kit—Cell and Plant (Exiqon‐Qiagen), was added to the column and centrifuged according to the exoEasy Maxi Kit instructions. The EV lysates were used immediately for RNA extraction following the protocol of the mirCURY RNA isolation kit—Cell and Plant (Exiqon‐Qiagen). EVs were isolated using the exoEasy kit based on its superior RNA yield and quality compared to ultracentrifugation, as confirmed by our pilot experiment and supported by previous studies [22, 23].

### 2.6 Cells and EVs RNA Extraction

Cells and EV RNA were extracted using the mirCURY RNA isolation kit‐Cell and Plant according to the manufacturer's instructions with modifications. The kit allows the isolation of total RNA and small RNAs, including miRNAs. Basically, the alteration was the exchange of the collection tubes for new ones after every centrifugation and washing steps to avoid reagent contamination of the EV RNA. RNAs were eluted with 50 μL of nuclease‐free water. RNA concentration was determined by the fluorometer Qubit (Thermo Fisher Scientific, Waltham, MA, USA) using the Qubit RNA HS Assay Kit (Thermo Fisher Scientific). Cell and EV RNA were analyzed on an RNA 6000 Pico chip using a 2100 Agilent Bioanalyzer (Agilent Genomics, Santa Clara, CA, USA) according to the manufacturer's protocol. EVs were isolated using the exoEasy Maxi Kit, which outperformed ultracentrifugation in RNA yield and small RNA profile quality (Figure S1A). Although we did not perform NTA in this study, the kit has been validated in previous studies, including by our group, for efficient EV recovery and RNA enrichment, supporting its suitability for our experimental goals.

### 2.7 miRNA Expression Profiling

miRNA profiling was performed using quantitative real‐time PCR (RT‐qPCR) with the pre‐configured TaqMan Low Density Array (TLDA) microfluidic cards (TaqMan Human MicroRNA Arrays Set v3.0, Applied Biosystems, Carlsbad, CA, USA). The array allows the detection of a total of 754 specific miRNAs and four control assays (RNU44, RNU48, and U6 as candidate endogenous controls and one negative control) distributed in two cards, Human Card A v2 and Human Card B v3. cDNA synthesis, pre‐amplification, and RT‐qPCR were performed as described in the protocol associated with the TaqMan Human MicroRNA Arrays Set v3.0. Briefly, 10 ng of RNA was reverse‐transcribed using Megaplex RT Primers, Human Pool A v2.1 and Megaplex RT Primers, Human Pool B v3.0. Following the manufacturer's recommendations for optimal sensitivity, a pre‐amplification step was included. Megaplex PreAmp Primers, Human Pool A v.2.1 and Megaplex PreAmp Primers, Human Pool B v3.0 were used for the pre‐amplification of the product of the reverse transcription reaction. The pre‐amplification product was diluted and used for the PCR mix preparation and loaded onto the TaqMan A or B microfluidic cards as described by the manufacturer. The arrays were run on a QuantStudio 7 Flex Real‐Time PCR System (Thermo Fisher Scientific), containing the Low Density Array Thermal Cycling Block installed. Pre‐processing of raw TLDA data files consisted of threshold corrections for each target. For each microRNA assay on TLDA cards, the threshold was set manually but consistently across all samples to ensure comparability. Amplification plots for each miRNA were individually inspected, and thresholds were adjusted to exclude aberrant curves while maintaining uniformity in data processing. The raw TLDA data were pre‐processed with threshold corrections applied equally across all samples for each target. Data normalization was performed using a global normalization strategy based on 87 assays consistently detected across all samples.

### 2.8 Data Processing and Analysis of TaqMan Human miRNA Arrays

The amplification plot of each miRNA was checked individually, and the threshold was set manually to exclude evident odd curves. To avoid technical and sample variability for low‐abundant miRNAs (*C*_*q*_ > 30) [24] and to obtain more reliable data, we set *C*_*q*_ = 30 as a cut‐off for detected miRNAs. We defined miRNAs with *C*_*q*_ < 28 in a group and *C*_*q*_ > 30 in another group as a situation where they were ‘expressed’ or ‘non‐expressed’ in that given comparison.

Assays with *C*_*q*_ values between 15 and 30 amplification cycles were considered expressed. Raw qPCR data were normalized by global normalization, including 87 assays detected in all samples [25]. Assays with one or more failed reads per group were arbitrarily discarded, so that means were calculated based on measures of the three biological replicates. Differentially expressed miRNAs between sample groups were determined with the R/Bioconductor package LIMMA [26, 27] (Linear Models for Microarray Data), a robust method specifically designed for high‐dimensional data such as expression arrays. miRNAs presenting less than 28 amplification cycles in one group and not detected in the other were considered expressed or non‐expressed. Statistical significance threshold was defined as adjusted *p*‐value ≤ 0.05 and absolute fold change (FC) ≥ 1.5. miRNA gene targets were found with the MIRWALK program using an experimentally validated database [28]. Over‐representation analyses were performed with the REACTOMEPA R/Bioconductor package [29]. Protein–protein interaction networks were built with up‐ and down‐regulated genes using the NETWORKANALYST program [30] and an experimentally validated database [31]. The network was constructed based on the subset of validated target genes specifically associated with membrane trafficking pathways. These core targets are highlighted in the network with blue‐bordered circles. Additional nodes represent first‐order interactors of these targets based on high‐confidence PPI data, though they are not directly annotated within canonical membrane trafficking pathways.

### 2.9 Target Validation by RT‐PCR

Total extracted RNA (1 μg) from three independent batches of cultured cells was used for reverse transcription with the SuperScript VILO cDNA Synthesis Kit (Thermo Scientific), according to the manufacturer's instructions. RT‐qPCRs were performed using PowerUp SYBR Green Master Mix (Thermo Scientific) with 25 ng of cDNA per reaction, using the StepOnePlus Real‐Time PCR Systems (Thermo Scientific). The RT‐qPCR cycle consisted of 95°C for 2 min, followed by 40 cycles of 95°C for 3 s and 60°C for 30 s. Beta‐actin (ACTB) and beta‐glucuronidase (GUSB) were used as reference mRNAs. For the quantification of target mRNAs by RT‐qPCR, we employed the comparative ΔΔCt method to calculate relative expression levels. Normalization was performed using the average of the two housekeeping genes ACTB and GUSB, selected based on their stable expression across our experimental conditions. The average was calculated using the arithmetic mean of their Ct values. The sequences of primers used for gene expression analysis are listed in Table S1.

### 2.10 Quantification and Statistical Analysis

Target validation by RT‐qPCR was based on data expressed as mean ± SEM, with individual dots representing the fold change average from three independent experiments per sample. For the analysis of mRNA expression, a limited number of genes were evaluated. After log transformation to approximate normal distribution, data were analyzed using parametric tests (e.g., unpaired *t*‐test or ANOVA, as appropriate). Multiple comparisons were corrected using the Holm‐Sidak method, with significance set at *α* = 0.05. Each comparison was analyzed individually, without assuming a consistent standard deviation. For miRNA profiling by qPCR, which involved high‐throughput detection of hundreds of miRNAs, data were normalized using the global mean of all reliably detected miRNAs per plate, a method shown to reduce technical variability in the absence of stable endogenous controls. Differential expression analysis was then performed with correction for multiple testing to control the false discovery rate (FDR).

## 3 Results

### 3.1 Differentiation of Human iPSCs Into Neural Stem Cells and Floor Plate Progenitors Followed by EV Isolation

**FIGURE 1:**
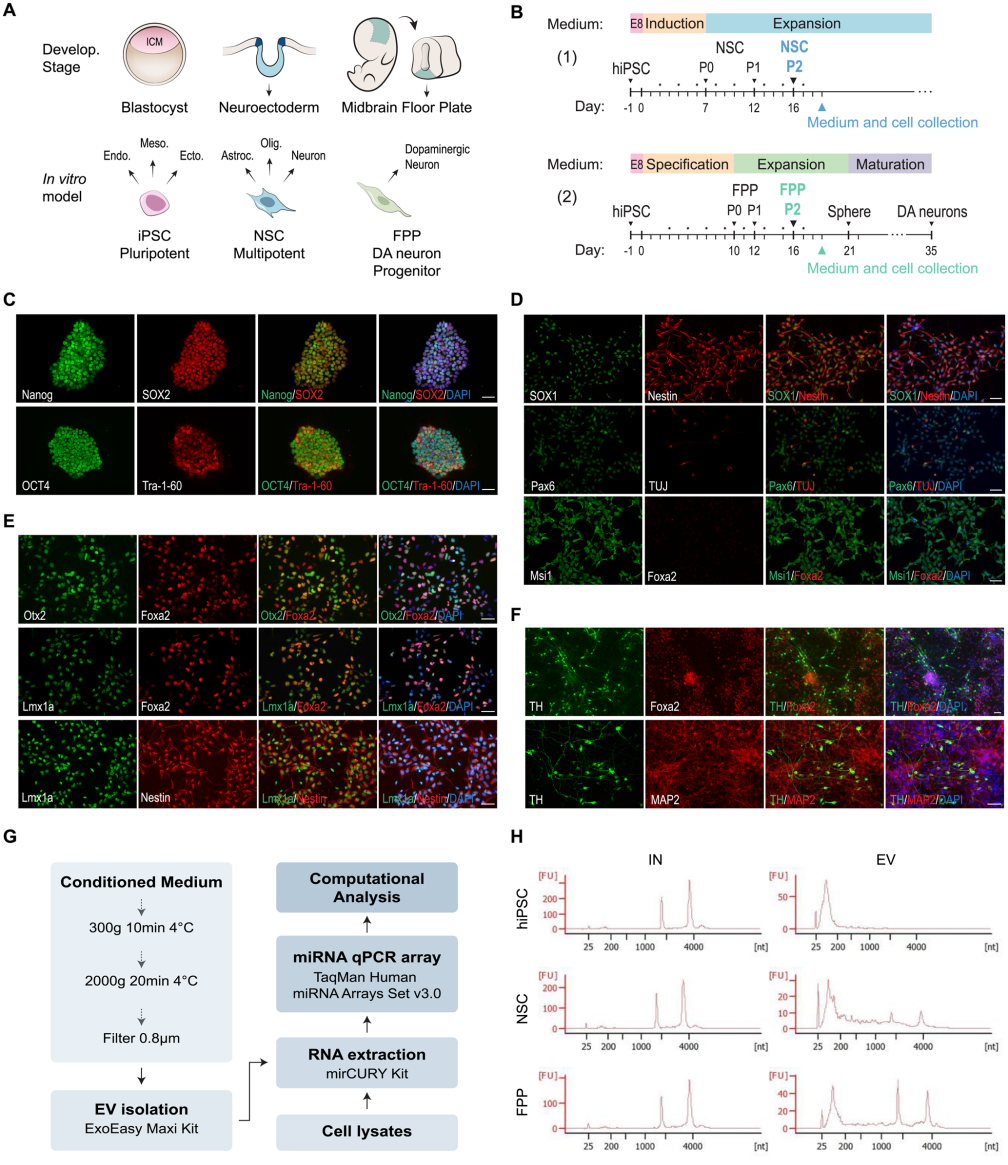
Design and characterization of experimental approach. (A) Equivalent developmental stages of the in vitro cell model. Induced pluripotent stem cells (iPSCs) can be differentiated into three germ layers: endoderm (Endo.), mesoderm (Meso.), and ectoderm (Ecto.). Neural stem cells (NSCs) are multipotent and can originate astrocytes (Astroc.), oligodendrocytes (Olig.), and neurons. Floor plate progenitors (FPP) can give rise dopaminergic neurons. ICM, inner cell mass. (B) Protocol of NSC (1) and FPP (2) differentiation. Passage numbers are indicated as P0–P2. Dots represent medium exchange. The medium was conditioned for approximately 48 h for EV isolation. (C) Immunofluorescence (IF) of pluripotency markers in hiPSC. (D) IF of neuroectodermal markers in NSC. (E) IF of FPP markers in FPPs. (F) FPPs were differentiated into dopaminergic neurons (tyrosine hydroxylase, TH^+^ neurons). Scale bars: 10 μm. (G) Experimental workflow. (H) Representative intracellular (IN) and EV RNA profiles assayed in Bioanalyzer. FU, fluorescence units; Nt, nucleotides.

To model early stages of neural differentiation, hiPSCs were differentiated into NSCs and FPPs (Figure 1A). Three hiPSC lines from different healthy donors (biological replicates) were maintained in the pluripotent state with the commercially available E8 medium. NSCs and FPPs were independently generated from hiPSCs using two distinct differentiation kit protocols consisting of their respective ‘Induction medium’ and ‘Expansion medium’ (Figure 1B). Under the defined culture conditions, hiPSCs were maintained indefinitely, while NSC and FPP expansion could be continued for up to 5 passages with a homogenous population. To provide validation of the pluripotency and the differentiation protocols, immunofluorescence assays were performed using typical markers expressed in hiPSCs (Figure 1C; Nanog, Sox2, Oct4, TRA‐1‐60), NSCs (Figure 1D; Sox1, Nestin, Pax6, Msi1), and FPPs (figure 1E; Lmx1a, Foxa2, Otx2) [4, 32]. To determine the capacity of FPPs to generate dopaminergic neurons, the protocol was continued through neurospheres and neuronal maturation until day 35, when the presence of MAP2 neurons positive for tyrosine hydroxylase (TH), a dopaminergic neuronal marker (Figure 1F) [4], was confirmed. The cell‐type‐specific marker confirmation demonstrated consistent and high differentiation efficiency for all three cell lines.

Cell‐conditioned media were collected according to each cell population's maintenance protocol (Figure 1B), centrifuged, and filtered before storage to completely remove cell fragments and vesicles sized below 0.8 μm. EVs from conditioned media were isolated using the commercially available exoEasy Maxi kit, based on a membrane affinity spin column method (Figure 1G). This kit allows selection of RNA present in EVs, since vesicle‐free RNA carried by protein complexes and lipoproteins is detected in the column flow‐through, which was discarded in this study [21, 33, 34]. To select the most suitable EV isolation method for downstream RNA analysis, we performed a pilot comparison between ultracentrifugation (2 h at 100 000 *g*) and the exoEasy kit using equal volumes of hiPSC‐conditioned media (32 mL). The exoEasy method yielded a significantly higher RNA concentration (43.5 vs. 10.7 ng/μL) and showed a more defined small RNA peak in the Bioanalyzer profile (Figure S1A). Based on these results, we adopted the column‐based exoEasy method for all subsequent steps. This choice is further supported by previous studies showing that exoEasy provides EVs with appropriate size, morphology, and protein profiles while minimizing contamination with non‐vesicular material. Additionally, functional assays using exoEasy‐isolated EVs have demonstrated preserved biological activity, reinforcing the reliability of the method for our experimental system (see the Material and methods section for details).

hiPSCs were the cell type with the highest RNA yield per mL of conditioned media, while FPPs were the cell type with the lowest RNA yield per mL (Figure S1B). This notable contrast can be attributed to the different maintenance conditions, cell confluency, amount of EVs released by each cell population, or their pluripotency/differentiation status. We next assessed the EV RNA size distribution profiles using a Bioanalyzer, and the traces showed distinct profiles depending on the cell population (Figure 1H). As previously observed, a dominant peak (25–200 nt) was consistently present in EV RNA preparations from all hiPSC lines, which corresponded to the small RNAs known to be enriched in EVs [33]. RNA extracted from NSCs and FPPs EVs also presented the expected small RNA peak, but showed two additional sizes that correspond to the ribosomal RNA subunits 18S and 28S. Interestingly, the ribosomal RNA (rRNA) peaks were more prominent in FPPs as compared to NSCs and did not appear in hiPSCs EVs, suggesting that rRNAs are selectively released in EVs according to the neuronal commitment (Figures 1H and S1C,D).

### 3.2 Profiling of Intracellular and EVs miRNA of hiPSCs, NSCs, and FPPs

**FIGURE 2:**
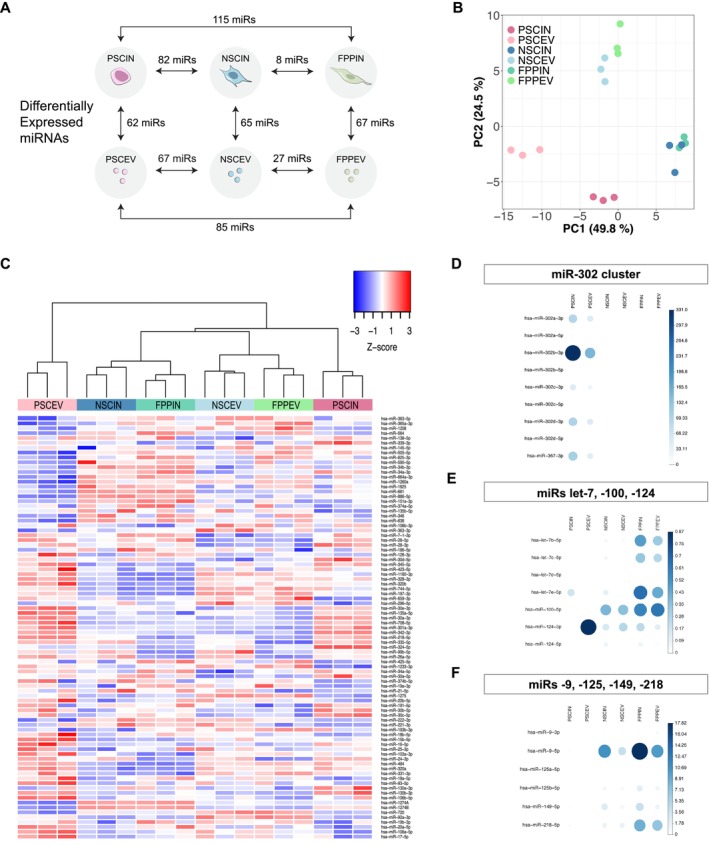
miRNA profiling distinguishes cells and EV populations. (A) Number of differentially expressed miRNAs (fold change > 1.5 and *p* < 0.05). Intracellular (IN) miRNAs from pluripotent stem cells, neural stem cells, and floor plate progenitors were named PSCIN, NSCIN, and FPPIN, respectively. Accordingly, the extracellular vesicle miRNA groups were named PSCEV, NSCEV, and FPPEV. (B) PCA clustered neural cells (NSCIN and FPPIN) apart from PSCIN, same pattern observed for PSCEV versus NSCEV and FPPEV. EV miRNA contents were distinct from their cells of origin (PSCEV, NSCEV, and FPPEV grouped apart from PSCIN, NSCIN, and FPPIN). (C) Heat map with the 87 differentially expressed miRNAs in all 18 samples. (D) Expression level of pluripotency miRNAs (miR‐302 cluster) and (E, F) neural differentiation‐associated miRNAs.

To determine the intracellular miRNA profile in dopaminergic neural differentiation and how the miRNA released through EVs reflected the pluripotency and neural commitment, we used a quantitative PCR (qPCR) TaqMan array (*TaqMan Array Human microRNA cards*), which covered a total of 754 miRNAs based on Sanger miRBase v14 (Figure 1G). We evaluated the intracellular (IN) miRNA profile of three hiPSCs (biological triplicates), their NSCs and FPPs differentiated cell populations (PSCIN, NSCIN, and FPPIN), and their respective EV miRNA profiles (PSCEV, NSCEV, and FPPEV) (Figure S2A). To explore the miRNA regulation differences between the groups, we determined the differentially expressed miRNAs (fold change > 1.5 and *p* < 0.05 and also included expressed and non‐expressed miRNAs according to the criteria we established prior to the analysis) (Figure 2A). For both intracellular and EV content, we found a higher number of miRNAs that distinguished the pluripotent state (PSC) from neural states (NSC and FPP) and a smaller number of miRNA differences between the neural populations (NSC and FPP). Of note, the number of differentially expressed miRNAs between intracellular and respective EVs was very similar within the groups (Figure 2A). On average, 26.3% and 24.1% of the assessed miRNAs (in the qPCR TaqMan array) were detected in the intracellular compartment and EVs, respectively (only miRNAs with Ct < 30 in the triplicate were considered) (Figure S2B). A total of 87 miRNAs (11.5%) were amplified in all 18 (intracellular and EV) samples simultaneously. Using these consistently expressed miRNAs, the heat map and principal‐component analysis (PCA) showed a clear cluster of the biological replicates within each group (Figure 2B,C). Taken together, these initial findings indicate that the qPCR array combined with the criteria established to analyze the miRNA expression is an appropriate approach to determine miRNA profiles in these human cell lines.

Previous studies have demonstrated a role for several intracellular miRNAs as key regulators of pluripotency state and neural differentiation [35, 36]. Here, we explored for the first time and compared with hiPSCs and NSCs, potential changes in the miRNA profile of FPPs. Remarkably, the PCA plot revealed that NSCs and FPPs were closely clustered, while hiPSCs were positioned further from neural lineages (Figure 2B). This distribution confirms that the pluripotent intracellular miRNA repertoire is clearly segregated from neural miRNA profiles and that FPPs miRNA content is very similar to that of NSCs (Figure 2B,C). EV samples clustered further from their respective intracellular samples, indicating distinct miRNA content expression. The EV sample distribution followed a similar pattern observed for intracellular: neural EVs separated from PSC EVs (Figure 2B,C). This suggests that the overall content of miRNAs in EVs reflects the intracellular differences among different cell types. In addition, our data analysis detected the expected changes in intracellular miRNA expression for markers of pluripotency/stemness (miR‐302/367 cluster, Figure 2D) or neural differentiation (let‐7 family, miR‐100, miR‐124, Figure 2E and miR‐9, miR‐125, miR‐149, miR‐218, Figure 2F) [37]. A clear decrease in miRNA expression related to pluripotency and an increase in neural fate‐associated miRNA were observed with neural commitment (Figure 2D). The majority of these markers followed the same pattern in the corresponding EV groups (Figure 2D–F). Altogether, these results indicate that our experimental and analytic approaches replicated well‐characterized miRNA expression patterns, providing high‐quality controls for our further miRNA comparative analysis.

**FIGURE 3:**
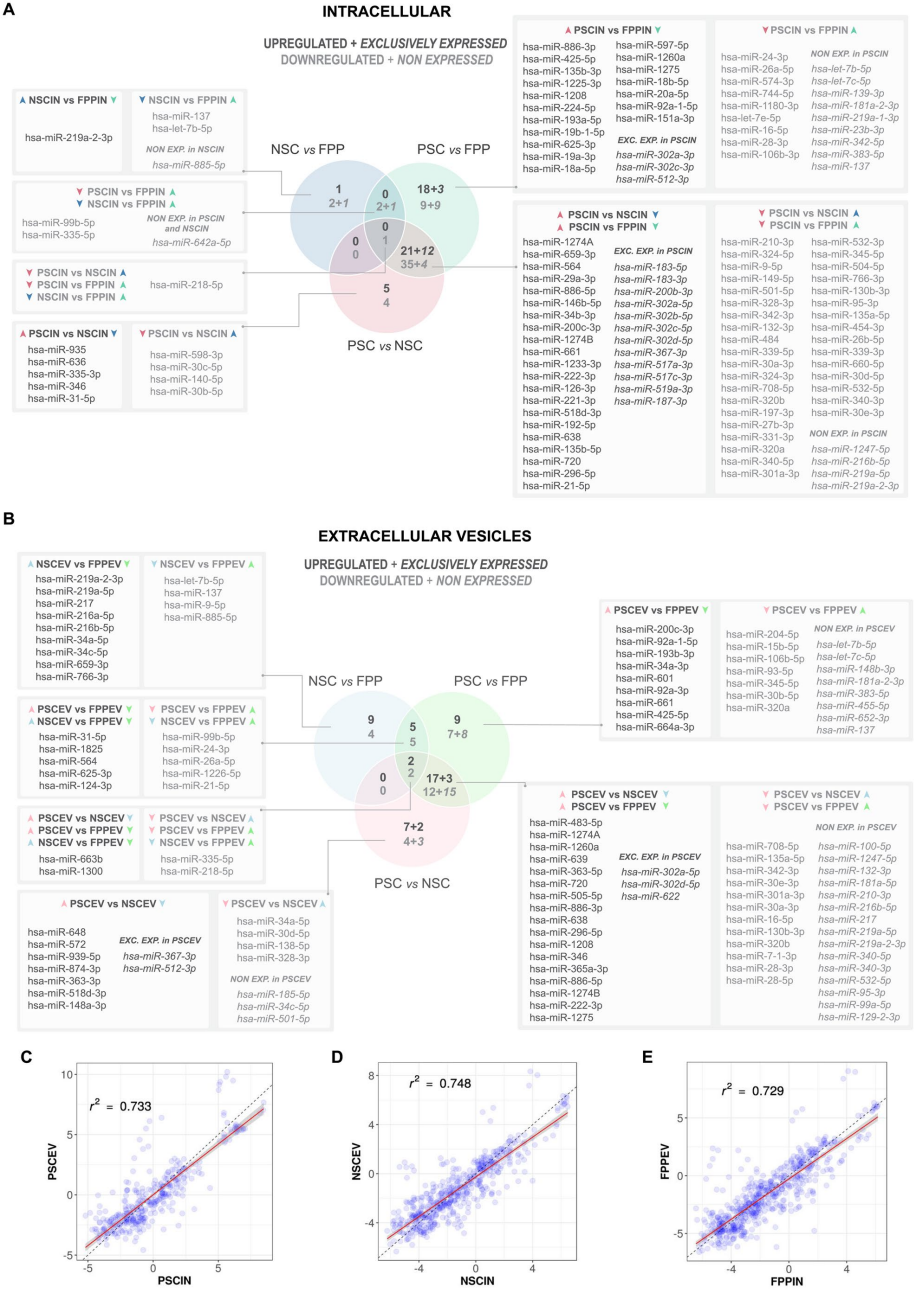
EVs' miRNAs correlate positively with their intracellular expression. (A, B) Venn diagrams of group comparisons in cells (A, intracellular) and in EVs (B, extracellular vesicles). For each comparison, a miRNA is upregulated or exclusively expressed (dark gray), downregulated or non‐expressed (light gray) in the first group compared to the second group. Differentially expressed miRNA IDs are displayed for each group comparison. Exclusively expressed and non‐expressed numbers and IDs of miRNAs in each comparison are represented in *italics*. miRNAs presenting less than 28 amplification cycles in one group and not detected (*C*_*q*_ > 30) in the other were considered expressed or non‐expressed. (C–E) EV miRNA expression displays a positive correlation with intracellular miRNA expression in all three developmental stage groups. Scatter plot of all miRNAs (*n* = 754), indicating intracellular miRNAs in the *x* axis and EVs miRNAs in the *y* axis for each group (C, PSC, D, NSC, and E, FPP). Arrow colors: Pink, blue, and green arrows represent miRNAs differentially expressed in pluripotent cells, neural stem cells, and floor plate progenitors, respectively. Up and down arrows indicate the direction of regulation. Dark‐colored arrows denote intracellular miRNAs, while light‐colored arrows correspond to EV‐associated miRNAs.

We next analyzed the identity of the differentially expressed miRNAs in Venn diagrams for comparisons among cell lines in intracellular compartments (Figure 3A) and EVs (Figure 3B). Changes are expressed as: (1) miRNA up or downregulated or (2) expressed or non‐expressed in which miRNAs were detected (Cts < 30) in only one experimental condition of a given comparison. According to previous studies, miRNAs released in EVs are correlated with their intracellular expression [38, 39]. Our data corroborated these previous findings, showing a positive correlation in all three cell populations (Figure 3C–E). In addition, by listing the 20 miRNAs with the highest levels in the EVs and the 20 miRNAs with the highest levels in the intracellular groups, we noted that the majority of miRNAs are the same between EVs and cells, with few exceptions (Figure S2C). This demonstrates that most miRNAs released in EVs reflect their intracellular levels in all three cell populations.

### 3.3 Differences in Intracellular miRNAs Between NSCs and FPPs

**FIGURE 4:**
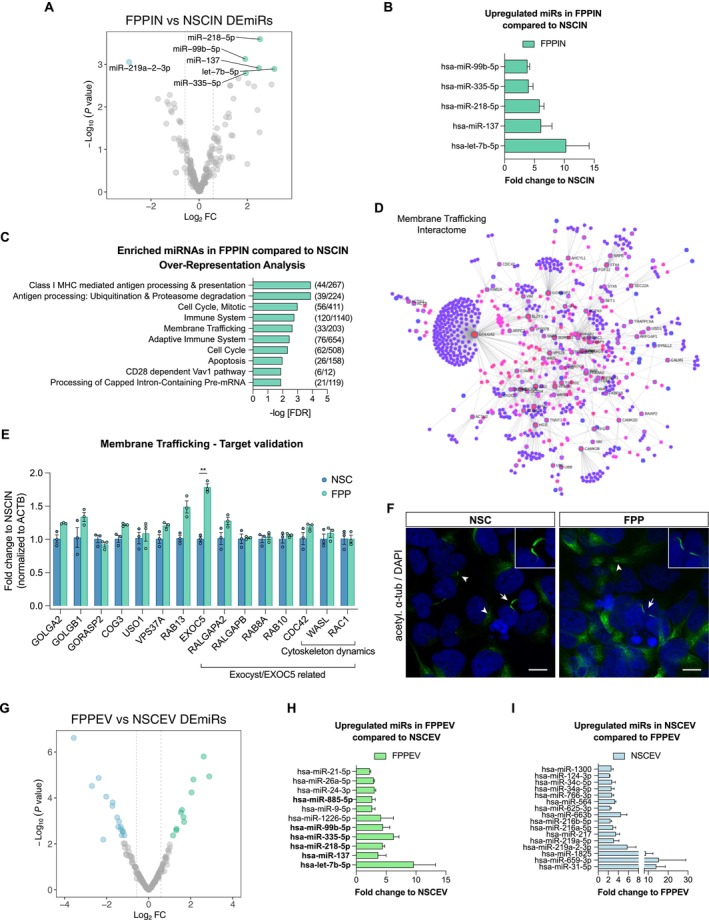
Intracellular and EV miRNA differences between FPPs and NSCs. (A) Volcano plot comparing NSC and FPP intracellular miRNAs profile. Vertical lines indicate fold change (FC) cut‐off of ±1.5. Differentially expressed miRNAs (DEmiRs) with adjusted *p*‐value ≤ 0.05 were highlighted (green are miRNAs more expressed in FPPs compared to NSCs, and blue, miRNAs more expressed in NSCs compared to FPPs). (B) Fold change of upregulated intracellular miRNAs in FPPs compared to NSCs. (C) Predicted targets from upregulated and expressed only in FPPIN miRNAs were used for pathway analysis. The top 10 pathways resulting from over‐representation analysis (ORA) are shown, and Membrane Trafficking was selected for further analysis. (D) Target interaction network from the Membrane trafficking pathway. Validated target genes (interaction score > 0.95) of differentially enriched EV‐miRNAs were identified using miRWalk 2.0 and used for pathway enrichment. A protein–protein interaction (PPI) network was constructed in NetworkAnalyst based on targets associated with membrane trafficking. Core targets are marked with blue‐bordered circles; additional nodes represent high‐confidence first‐order interactors. (E) Representative hubs from network (D) were selected for target validation by qPCR in the same biological replicate used in the array (in three independent experiments). EXOC5 showed statistically significantly different expression between groups (adjusted *p* value = 0.0018). Exocyst/EXOC5‐related targets were also tested. Data are represented as mean ± SEM. Each dot represents the fold change average of three independent experiments for each sample. Multiple *t*‐tests corrected for multiple comparisons with the Holm‐Sidak method. Statistical significance determined using the Holm‐Sidak method, with alpha = 0.05. Each row was analyzed individually, without assuming a consistent SD. (F) Immunolabeling of acetylated alpha‐tubulin in NSCs and FPPs. Evidence of single primary cilia (arrow heads) and primary cilia pointing to each other between two cells (arrows and crop on top right). Staining was performed in all three biological samples, and representative images are shown. Nuclei were counterstained with DAPI. Images were acquired using the Zeiss confocal system. Scale bars: 10 μm. (G) Volcano plot comparing NSC and FPP EV miRNA profile. Vertical lines indicate fold change (FC) cut‐off of ±1.5. Differentially expressed miRNAs (DEmiRs) with adjusted *p*‐value ≤ 0.05 were highlighted (green are miRNAs more expressed in FPP EVs compared to NSC EVs, and blue, miRNAs more expressed in NSC EVs compared to FPP EVs). Volcano plots were generated using identical cut‐offs for adjusted *p*‐value and fold‐change. The difference in *y*‐axis scales (0–2 in 4A; 0–6 in 4G) reflects dataset‐specific visualization needs. (H) Fold change of upregulated EVs miRNAs in FPPs compared to NSCs. miRNAs in bold indicate the same ones identified in the intracellular comparison. (I) Fold change of upregulated miRNAs in EVs in NSCs compared to FPPs. Data are represented as mean ± SEM.

To investigate miRNA regulatory differences between multipotent neural progenitor cells (NSCs) and dopaminergic neuron‐committed progenitor cells (FPPs), we aimed to identify both the differentially expressed miRNAs and the pathways associated with their predicted targets (Figures 3A and 4). We found five upregulated (hsa‐miR‐218‐5p, ‐335‐5p, ‐137, ‐let7‐5p, and ‐99b‐5p) miRNAs in FPPs when compared to NSCs and two miRNAs expressed only in FPPs (hsa‐miR‐885‐5p and ‐642a‐5p) (Figure 3A). The only downregulated miRNA in FPPs, when compared to NSCs, was hsa‐miR‐219a‐2‐3p (Figures 3A and 4A). Overrepresentation analysis (ORA) with the predicted mRNA targets of the miRNAs, which were upregulated and exclusively expressed in FPPs, revealed multiple pathways, including membrane trafficking (Figure 4C), a particularly interesting pathway since it has gained special attention as the underlying etiology of PD [40]. We next sought to validate this pathway and performed interaction network analyses with members of membrane trafficking pathways, which also included the targets of the differentially expressed miRNAs (Figure 4D). Potential changes in mRNA expression of targets related to the hubs from this network were tested using RT‐qPCR in the same samples used in the array. Exocyst complex component 5 (EXOC5 or Sec10) was validated as an upregulated gene in FPPs compared to NSCs (Figure 4E). Although miR‐218‐5p was upregulated in FPPs compared to NSCs, its predicted target EXOC5 was also found to be upregulated. This apparent discrepancy may reflect complex regulatory interactions, such as compensatory transcriptional activation, reduced miRNA‐mediated repression due to competing endogenous RNA competition, or temporal mismatches between transcriptional and post‐transcriptional regulation. These findings highlight the need for functional validation beyond correlative expression data. From the FPP miRNAs found in the analysis, EXOC5 mRNA is a target of hsa‐miR‐218‐5p and ‐642a‐5p. EXOC5 is a member of the exocyst complex implicated in the targeting of exocytic vesicles to specific docking sites on the plasma membrane [41] and is critical in determining the morphology and function of primary cilia [42], an essential organelle for FPP‐mediated SHH signaling in vivo [43, 44]. We also selected targets that were directly related to exocyst/EXOC5 and verified their expression levels by RT‐qPCR (Figure 4E). We next showed evidence of the primary cilium in both NSCs and FPPs by acetylated alpha‐tubulin immunolabeling. The primary cilium projects as a single organelle from the surface of these cells (arrow heads, Figure 4F), and in between some cells, they presented facing each other (arrows, Figure 4F). To complement these intracellular findings and explore intercellular communication, we also examined pathways related to the 27 differentially enriched miRNAs associated with EVs released by FPPs (11 upregulated miRNAs in FPPEV vs. NSCEV) and NSCs (16 upregulated miRNAs in NSCEV vs. FPPEV) (Figure S3A,B). Pathway enrichment analysis of their predicted targets revealed that FPP‐derived EVs were enriched in miRNAs targeting transcriptional activity, cell cycle progression, and mitogenic signaling, consistent with the downregulation of proliferative programs as cells transition into a more differentiated state. In contrast, NSC‐derived EVs carried miRNAs targeting pathways critical for early developmental regulation, including TGF‐β signaling, receptor tyrosine kinase cascades, SUMOylation, and transcriptional control. The reduced abundance of these miRNAs in FPP‐EVs may reflect de‐repression of these pathways, potentially enabling lineage specification and decreased stemness during neural commitment (Figure S3A,B). Together, these data suggest that the intracellular and EV‐associated miRNA signatures of FPPs act in parallel to reinforce cell fate decisions and modulate cellular machinery, including membrane trafficking processes, which may be relevant to the selective vulnerability of dopaminergic neurons in PD.

### 3.4 FPPs Differ From NSCs More in Their EVs Than in Their Intracellular miRNA Content

In the developing brain, FPPs are positioned in the ventral region with cells facing the lumen of the neural tube, suggesting a cellular polarization with high secretory capacity [45, 46]. Indeed, in vitro midbrain FPPs secrete functional morphogens, which are closely related to their role in the patterning of the surrounding tissue and neuronal guidance [6, 47]. Moreover, midbrain FPPs also possess the capacity to give rise to dopaminergic neurons [4]. Because of this secretory property, we hypothesized that FPPs exert their developmental modulatory function by releasing molecular factors through EVs, including miRNAs. To this end, we asked whether FPP EVs are enriched in certain miRNAs that segregate them from NSC EV content. Indeed, the comparative analysis showed that the number of miRNAs differentially expressed between NSC and FPP is higher in EVs than in their intracellular compartments (Figures 2A and 4G–I).

From the 11 upregulated miRNAs in FPP EVs compared to NSC EVs, 6 were the same miRNAs either expressed at higher levels or exclusively expressed in the intracellular profiles of FPPs (hsa‐let‐7b‐5p, miR‐137, ‐218‐5p, ‐335‐5p, ‐99b‐5p, and ‐885‐5p; Figure 4H). Thus, more than 50% of the miRNAs carried by FPP EVs that distinguish them from NSC EVs reflect their intracellular differences. The other five upregulated miRNAs in FPP EVs compared to NSC EVs are hsa‐miR‐21‐5p, ‐26a‐5p, ‐24‐3p, ‐9‐5p, and ‐1226‐5p (Figure 4H).

**FIGURE 5:**
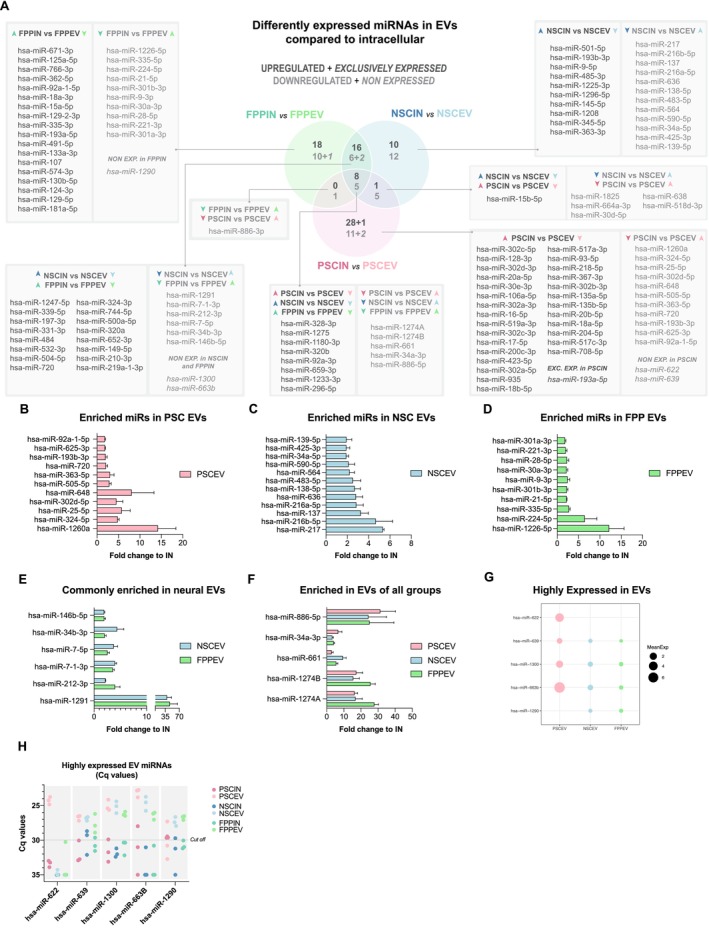
Extracellular versus intracellular miRNA expression pattern reveals EV miRNA signatures. (A) Venn diagram of intracellular versus EV miRNAs comparisons. For each comparison, a miRNA is upregulated or exclusively expressed (dark gray) and downregulated or non‐expressed (light gray) in the intracellular group compared to its corresponding EV group. Exclusively expressed and non‐expressed numbers and IDs of miRNAs in each comparison are represented in *italics*. miRNAs presenting less than 28 amplification cycles in one group and not detected (*C*_*q*_ > 30) in the other were considered expressed or non‐expressed. (B–G) Fold change of miRNAs enriched in EVs. Fold change of miRNAs enriched specifically in EVs of pluripotent stage cells (B), multipotent neural stage cells (C), and dopaminergic neuronal committed progenitor cells (D). (E) Fold change of miRNAs enriched in EVs of both neural populations (NSCEV and FPPEV). (F) Fold change of miRNAs enriched in all EVs independent of developmental stage. (G) Expression level of miRNAs highly expressed in EVs. (H) *C*_*q*_ values of miRNAs highly expressed in EVs. Data are represented as mean ± SEM. Arrow colors: Pink, blue, and green arrows represent miRNAs differentially expressed in pluripotent cells, neural stem cells, and floor plate progenitors, respectively. Up and down arrows indicate the direction of regulation. Dark‐colored arrows denote intracellular miRNAs, while light‐colored arrows correspond to EV‐associated miRNAs.

Among all 11 miRNAs upregulated in FPP EVs compared to NSC EVs, only three (miR‐21‐5p, ‐1226‐5p, and ‐335‐5p) were found in higher levels in FPP EVs than in the FPP intracellular compartment (Figure 5A). The other eight FPP EV‐enriched miRNAs (compared to NSC EVs) were found at the same level of expression as their intracellular miRNAs (FPPIN). Considering the upregulated miRNAs in NSC EVs, the analysis detected 16 miRNAs, which were more highly expressed in NSC EVs compared to FPP EVs (Figure 4I). Among these 16 miRNAs, only hsa‐miR‐219a‐2‐3p was also found to be upregulated in NSCs' intracellular content when compared to FPPs (Figure 3A). Eight of the 16 miRNAs upregulated in NSC EVs compared to FPP EVs were found in higher levels in NSC EVs than in their intracellular compartment (NSCIN) (Figure 5A). Those were hsa‐miR‐217, ‐216a‐5p, ‐216b‐5p, ‐34a‐5p, ‐1825, ‐564, ‐663b, and ‐1300. This indicates that half of the upregulated miRNAs in NSC EVs compared to FPP EVs were selectively released in EVs from NSCs. Overall, these data suggest that differentially expressed EV miRNAs may represent major extracellular functional differences between these neural cell populations.

### 3.5 Extracellular Versus Intracellular miRNA Expression Patterns

We then compared cellular content to their secreted EVs profile to identify the differential enrichment of specific miRNAs in EVs and whether this aspect could distinguish each population (Figure 5A). The fold change of miRNAs more highly expressed in EVs than their cell of origin is presented for PSCs (Figure 5B), NSCs (Figure 5C), and FPPs (Figure 5D). Each group had a set of around 10 miRNAs that were specifically upregulated in their respective EVs (Figure 5B–D). The hsa‐miR‐1260a in PSC EVs and hsa‐miR‐1226‐5p in FPP EVs were the most upregulated miRNAs in their groups, with average fold change > 10 (Figure 5B,D). Next, we asked whether neural population EVs shared enriched miRNAs that could distinguish them from the pluripotent EVs (Figure 5A). Among the six miRNAs detected in our analysis for this category, hsa‐miR‐1291 was the most upregulated miRNA in both NSC EVs and FPP EVs when compared to PSC EVs (Figure 5E). These findings imply that specific miRNAs are selectively secreted through EVs, which differ for each cell type. Those enriched miRNAs, together with other released miRNAs (and factors), could contribute synergistically to create a unique cell niche environment, with the potential to have an active influence on the development of different cell populations.

### 3.6 A Signature of miRNAs in EVs

We detected a set of miRNAs consistently enhanced in EVs in all three cell types. It included hsa‐miR‐1274A, ‐1274B, ‐34a‐3p, ‐661, and ‐886‐5p (Figure 5A,F). Among the consistently overexpressed EV miRNA, miR‐1274A, ‐1274B, and ‐886‐5p were higher in PSC EVs and PSC intracellular content when compared to the neural populations (Figure 3A,B). The analysis also detected some cases of miRNAs unique to EVs and low‐abundant or not expressed at detectable levels within cells, such as hsa‐miR‐622, ‐639, ‐1300, ‐663b, and ‐1290 (Figure 5A,G). These findings were based on our study criteria, in which a miRNA was considered expressed in a condition where its *C*_*q*_ value was *C*_*q*_ < 28 and *C*_*q*_ > 30 in the other group. Because we expect that miRNAs released in EVs would have some level of expression inside the cells, we describe this set of miRNAs based on their *C*_*q*_s and statistical analysis (Figure 5G). MiR‐622 is an exclusive miRNA of EVs from PSCs (mean *C*_*q*_ values: PSC EV = 24.3 and PSC IN = 33.4), being neither detected in EVs nor in cells from neural populations (mean *C*_*q*_ values > 35). While hsa‐miR‐639 is low abundant in intracellular compartments of all cells (*C*_*q*_ > 30), it was enriched in all EVs, with PSCs presenting the highest difference in the contrast EVs vs intracellular (mean *C*_*q*_ values: PSC EV = 26.8 and PSC IN = 31.9; NSC EV = 26.9 and NSC IN = 30; FPP EV = 27.7 and FPP IN = 30.7) (Figure 5G). Comparing its expression in the EV groups, hsa‐miR‐639 was upregulated in PSC EVs compared to neural EVs (Figure 3B).

We also evaluated the top 10 pathways regulated by miRNAs specifically enriched in each EV population (from Figure 5B–D), PSCEVs, NSCEVs, and FPPEVs, analyzed separately (Figure S3B). We found that PSCEV‐derived miRNAs predominantly targeted pathways such as TGF‐β signaling, Hippo signaling, and circadian rhythm regulation. In NSCEVs, enriched pathways included TGF‐β signaling, estrogen receptor beta (ERβ) signaling, and adherens junctions. In contrast, FPPEVs showed enrichment for miRNAs regulating pathways associated with prion diseases, Hippo signaling, and estrogen signaling, highlighting a dynamic shift in extracellular miRNA‐mediated signaling across neural differentiation.

MiR‐1300 was also low abundant in the cells and enriched in EVs (mean *C*_*q*_ values: PSC EV = 25 and PSC IN = 31.6; NSC EV = 25.2 and NSC IN = 31.9; FPP EV = 26.2 and FPP IN = 31). It was more enriched in NSC EVs and FPP EVs compared to NSC and FPP intracellular compartments but not statistically different in the PSC EVs vs PSC intracellular content contrast due to one PSC intracellular sample expressing it (*C*_*q*_ = 29.9). MiR‐663b was highly expressed in all EVs, with most of the intracellular samples not presenting detectable levels (mean *C*_*q*_ values: PSC EV = 23.1 and PSC IN = 33; NSC EV = 24.7 and NSC IN = 37; FPP EV = 26.4 and FPP IN = 36.9). Same as hsa‐miR‐1300, it was not identified as statistically different in the PSC EVs versus PSC intracellular content contrast due to one PSC replicate sample expressing it (*C*_*q*_ = 28). MiR‐1290 was enriched only in NSC EVs and FPP EVs, and it was low‐abundant in the intracellular compartments of the same groups. MiR‐1290 presented statistical differences only in FPP EVs compared to FPP intracellular content due to replicate variability in NSC intracellular samples (mean *C*_*q*_ values: PSC EV = 30.3 and PSC IN = 29.6; NSC EV = 27.3 and NSC IN = 33.6; FPP EV = 26.7 and FPP IN = 30.7) (Figure 5H). Altogether, these findings revealed a set of miRNAs either selectively released in EVs for all groups or presenting an advantageous stability among other miRNAs.

## 4 Discussion

This study is the first comprehensive characterization of miRNAs released by EVs from a pluripotent state to neural differentiated precursor cells, including the multipotent NSCs and the dopaminergic neuronal committed precursors. This will allow further studies on cell/stage‐specific EV miRNAs as extracellular regulatory signals during patterning. Here, we discuss some of the fundamental contributions of this work to the field.

### 4.1 Intracellular miRNA Profile

We identified five upregulated (hsa‐miR‐218‐5p, ‐335‐5p, ‐137, ‐let7b‐5p, and ‐99b‐5p) miRNAs in FPPs compared to NSCs and two expressed (has‐miR‐885‐5p and ‐642a‐5p) miRNAs specifically in FPPs. The only downregulated miRNA in FPPs compared to NSCs was hsa‐miR‐219a‐2‐3p. The pathway analysis performed with predicted targets of these seven upregulated/exclusively expressed miRNAs pointed to membrane trafficking as potential pathways regulated by those miRNAs in FPPs. The target validation of the protein interaction network related to membrane trafficking revealed an upregulation of EXOC5 (target of miR‐642a‐5p, ‐218‐5p). In this session, we provide evidence from previous studies to (1) support the difference in miRNA expression levels and (2) support a potential relationship between those miRNAs, their putative targets, and the FPP importance on dopaminergic differentiation and PD.

#### 4.1.1 About DE miRNAs Expression in FPP

Mir‐218 is important for the development of dopaminergic and motor neurons [48, 49]. Interestingly, miRs‐218 (−1 and −2) are located in introns of the *SLIT2* and *SLIT3* genes respectively, and Slit2, like netrin‐1, is both expressed in floor plate cells and is a secreted molecule with roles in axon guidance [6, 18, 50]. The miR‐218 co‐expression and co‐regulation with its host gene *SLIT2/3* have been shown mainly in tumor cells and motor neurons [51, 52]. MiR‐335 is located in an intron of the mesoderm‐specific transcript gene (*MEST*, or paternally expressed gene 1, *PEG1*), a paternally expressed imprinted gene that is highly expressed and essential for the development of midbrain dopaminergic neurons, among other functions [53, 54]. *MEST* was shown to be highly expressed in the midbrain floor plate area of the developing neural tube, and expression was sustained through the adult stage, specifically in dopaminergic neurons of the substantia nigra, the brain region affected in PD [54]. These observations strongly suggest that the upregulated miRNAs, miR‐335‐5p and miR‐218‐5p in FPPs identified in our analysis, are a result of the expression of their respective host genes *MEST* and *SLIT2/3*, which are known to be highly expressed in FPP. This indicates that essential genes for dopaminergic differentiation could lead to the co‐expression of their intronic miRNAs, which in turn can define modulatory post‐transcriptional programs relevant to specific features of dopaminergic neuronal progenitors.

The Let‐7 family has been widely studied in several physiological [35, 55, 56] and pathological processes [57, 58, 59]. The expression of the Let‐7 family is negatively regulated by the RNA‐binding protein LIN28 [60]. Notably, the LIN28‐let‐7 axis has been characterized during neural commitment, with some studies revealing that LIN28 controls the processing of the precursor pre‐let‐7 into its mature form in NSCs but not in hESCs (where the precursor form was ubiquitously expressed) [35, 56, 61]. This suggested let‐7 biogenesis and function are cell‐type specific. A recent study identified a loss‐of‐function mutation in LIN28A in PD patients and showed that conditional knockout of LIN28 in mice induced degeneration of midbrain dopaminergic neurons in the substantia nigra and PD‐related behavioral deficits [57]. Notably, there is evidence that neonatal neurogenesis, but not late adult neurogenesis, is implicated in the etiology of PD [62]. Let7 has been identified as one of the mitochondrial miRNAs (mitomiRs), which are located in mitochondria and have the potential to modulate mitochondrial activities [63, 64]. Indeed, mitochondrial dysfunction is a hallmark of PD pathogenesis [65]. A previous study indicated ATP2B2, ATP2B4, and ATP2C2 mRNAs as potential mitochondrial targets of let‐7b‐5p and let‐7a‐5p [66]. However, a role of LIN28/let‐7b‐5p in FPP and PD involving let‐7b activities in mitochondria has not been addressed so far.

Interestingly, miR‐885 (highly expressed in FPPs) is located in an intron of *ATP2B2* [67]. MiR‐885‐5p has been shown to be significantly overexpressed in the blood of PD patients [68, 69] and in SH‐SY5Y neuronal‐like cells overexpressing γ‐synuclein [70]. So far, miR‐99b‐5p has not been studied in dopaminergic differentiation or signaling, and miR‐137 has been shown to modulate synaptic plasticity, mitochondrial dynamics, dopamine transporter expression, and dopamine receptor (D2R) expression [71, 72, 73, 74].

#### 4.1.2 About miRNAs Predicted Targets

We identified EXOC5 upregulation in FPP compared to NSC, and EXOC5 mRNA is a predicted target of miR‐642a‐5p, which was also upregulated in FPPs. In the miRbase, other exocyst complex components appear as miR‐642a‐5p targets: EXOC3, EXOC6B, and EXOC8. In TargetScan, EXOC5 is predicted to be regulated by miR‐218‐5p (upregulated in FPPs). EXOC5 (Sec10) is a member of the exocyst complex, which is responsible for vesicle trafficking and docking to the cell membrane [41, 75]. RALGAPA2 (predicted target of miR‐335‐5p) is a member of the Ral family of GTPases, which contributes to exocyst complex assembly by interacting with Sec5 and Exo84 [76, 77]. Studies have shown that exocyst components are localized in primary cilia, which are short membrane projections, microtubule‐based organelles, found at the surface of many cell types [78, 79, 80]. Primary cilia exert specialized sensory and signaling functions, which involve Shh, Wnt, and FGF pathways during development and tissue homeostasis [81]. There is evidence that FPP effects on neural patterning are mediated by their primary cilia [43, 82, 83], which were shown to be longer than cilia presented by other neural progenitors [44]. Cilia formation and function were also shown to be affected by PD‐associated LRRK2 kinase/Rab GTPases, suggesting a contribution of primary cilia in PD‐specific pathology [84]. In renal cells, knockdown of EXOC5 results in very short or absent cilia, and EXOC5 overexpression results in longer cilia [42, 85, 86]. Moreover, primary cilia can be a source of bioactive EVs, named ectosomes [87, 88]. EXOC5‐containing vesicles were observed at the tip and sides of primary cilia, and EXOC5 expression was found to impact EV release and EV protein content in Madin‐Darby canine kidney (MDCK) cells [42, 86]. A link for miRNAs, EXOC5, and EVs integrated to the primary cilia importance in FPP biology is still missing.

### 4.2 Extracellular Profile

While we aimed to characterize miRNAs, our data also provide evidence for the presence of other RNA fragments that are no longer included in the miRbase. These included rRNA subunits and fragments of tRNA (miR‐1274, ‐1260, ‐886‐5p), rRNA (mir‐663, ‐1275), mRNA (miR‐1300), and vaultRNA (miR‐886) in the purified Ev RNA. These unexpected findings indicate a cell type‐specific EV signature that can be a consequence of the RNA metabolism status in each developmental stage.

The EV RNA profile revealed by the Bioanalyzer showed the presence of rRNA subunits 18S and 28S specifically in NSC Evs and FPP Evs, with an apparent cell stage‐dependent effect (peaks were more prominent in FPP Evs compared to NSC Evs). rRNA has been described in the sequencing of EV RNA from different cell types [89, 90]. Moreover, rRNA synthesis is regulated in response to metabolic and environmental changes, as well as during neural differentiation [91]. The NAD‐dependent histone deacetylase Sirtuin‐1 (SIRT1) negatively regulates rRNA processing in response to the metabolism of cells [92]. Interestingly, SIRT1 has been shown to be highly expressed in pluripotent stages and downregulated upon neural differentiation [93, 94]. Considering its repressive control of rRNA expression in response to cell metabolic status, we hypothesize that the presence of rRNA in EVs of the neural population could be a consequence of low expression of SIRT1, leading to higher rRNA synthesis and release. Our observation of cell dependent rRNA subunits 18S and 28S in EVs is supported by previous studies which showed using Bioanalyzer that although rRNA subunits were not seen in exosomes, their presence in microvesicle fractions was cell type dependent [95, 96, 97]. Based on that, our data suggest that EVs released by PSCs are preferentially composed of exosomes, while EVs from NSCs and FPPs are predominantly microvesicles. Potential changes in EV subtypes during neural differentiation require future investigation.

To identify miRNA enrichment in EVs, we compared miRNA expression in EVs with their respective source cells. A set of miRNAs was remarkably enriched in EVs (> 10 fold) and consisted of miR‐1260a, ‐622, ‐639 (PSC EVs); miR‐1226‐5p, ‐1290 (FPP EVs); miR‐1291, ‐1300, ‐663b (NSC EVs and FPP EVs); and miR‐886‐5p, ‐1274A, ‐1274B (PSC EVs, NSC EVs and FPP EVs). Most of these share a similar feature; they were identified as fragments of other RNA types, and indeed, some (i.e., miR‐1274A, ‐1274B, ‐1300, ‐886‐5p) have been excluded from the miRBase. MiR‐1274A, ‐1274B, ‐886‐5p, and ‐1260a were considered products of tRNA processing [98, 99]; miR‐886‐5p also as a product of the *vault* RNA (vtRNA2‐1) or non‐coding 886 (nc886) [100, 101]; miR‐1300, a fragment of the Elongation factor 1‐alpha (EEF1A) mRNA [102]; miR‐1291, derived from a small nucleolar RNA [103]; and miR‐663b, originated from rRNA [104]. Small ncRNAs like tRNA fragments (tRFs) have a stem‐loop hairpin structure, as do miRNAs precursors, and tRFs can originate from the hairpin stem, like mature miRNAs [99, 105]. Such similarities could lead to a misannotation of the nature of those fragments. In addition, like miRNAs, tRNAs can also have regulatory functions on mRNA expression and have been found to be abundant in EVs [106, 107, 108].

Our study revealed a unique EV miRNA/small RNA signature in each cell population. This means that not only intracellular miRNA content, but EV miRNA content can also distinguish these cell types. This is in part due to the fact that EVs mirror their cell of origin [38], but we also detected miRNAs that were more enriched in EVs than their cells of origin, increasing the differences among EVs more than their intracellular counterparts. Likewise, we showed that FPPs differ from NSCs more in their EVs than in their intracellular miRNA content. In addition to the upregulated intracellular miRNAs described in FPP intracellular content, miR‐21‐5p, ‐26a‐5p, ‐24‐3p, ‐9‐5p, and ‐1226‐5p were also found to be upregulated in FPP EVs when compared to NSC EVs. MiR‐21‐5p is a well‐characterized EV‐carrying miRNA in neural differentiation, as well as in neuronal and tumor communication [109, 110, 111]. Among these miRNAs, miR‐1226‐5p is not a commonly studied EV miRNA, and so far, it has been shown to be enriched in EVs of a colon cancer cell line [112]. Notably, miR‐1226 is a miRNA found in an intron of the *DHX30* gene and is also considered a mirtron, since it uses splicing to bypass Drosha cleavage [113]. DHX30 is an ATP‐dependent RNA helicase that participates in RNA metabolism, including in mitochondria, and has been implicated in a neurodevelopmental disorder with severe motor impairment and absence of language (NEDMIAL) [114, 115]. A correlation between DHX30 expression/function in FPP and its mirtron miR‐1226‐5p still remains to be elucidated.

### 4.3 Study Relevance

EVs have been considered relevant tools for therapeutic purposes due to their capacity to act as delivery vehicles for modulatory biomolecules [116, 117, 118]. Thus, EVs from the cells used in this work offer an alternative for cell therapy in regenerative medicine and neurodegenerative diseases, mainly because cell‐based therapy can present consequences that cannot be totally controlled [119]. Indeed, developmental processes are strictly controlled, and their failure can lead to clinical complications (developmental diseases) [120]. In addition, miRNAs have been described as biomarkers of pathological conditions [121, 122]; therefore, they are considered potential targets for therapies that can restore their normal levels. In this sense, this work provides additional information for molecular characterization of neurodevelopment. As an example, the miRNAs described in FPP EVs could be relevant for studies focused on the biology of FPPs beyond their role as a source of dopaminergic neurons. FPPs facing the neural tube present epithelium properties, along with the presence of primary cilia as signaling organelles. FPPs also act as a developmental organizing center due to the release of morphogens that affect neighboring cells. In this scenario, the differentially expressed miRNAs in EVs of FPPs could indicate some specific miRNA contribution in the FPP niche. Moreover, as miRNAs have been successfully used in cell reprogramming and differentiation methods [27, 123, 124]. This work provides resource data that can support the direct or indirect use of select miRNAs in protocols of dopaminergic neuron differentiation. Improvements in the protocols are essential due to their high implication in cell therapy applied to neurodegenerative diseases [125].

In summary, this study uncovers a set of candidate intracellular and extracellular miRNAs potentially involved in dopaminergic specification, providing novel insights into the molecular landscape of neural differentiation and EV‐mediated communication. While the findings are exploratory, they establish a strong framework for future functional validation and for the development of miRNA‐based strategies in regenerative neuroscience. These data may guide further investigations to improve in vitro disease modeling, advance our understanding of human neurodevelopment, and support drug discovery efforts. Ultimately, this work may contribute to cell therapy approaches for neurological disorders or enable targeted modulation of the neuronal microenvironment through the use of secreted miRNAs and phenotype‐specific EVs.

## Author Contributions

Conceptualization, L.C., F.M.F, C.M.L.‐R., W.T.H., Z.W., H.I.N., D.C.B., A.M.K., X.O.B., and M.H.L.; Methodology, L.C., F.M.F, C.M.L.‐R., W.T.H., Z.W., and H.I.N.; Formal Analysis, L.C., and F.M.F; Validation, L.C.; Investigation, L.C.; Resources, H.I.N., H.A.J., D.C.B., X.O.B., and M.H.L.; Writing – Original Draft, L.C. and M.H.L.; Writing – Review and Editing, F.M.F, C.M.L.‐R., W.T.H., Z.W., H.I.N., H.A.J., D.C.B., A.M.K., X.O.B., and M.H.L.; Visualization, L.C. and F.M.F; Supervision, A.M.K., X.O.B., and M.H.L.; Funding Acquisition, L.C., and M.H.L.

## Conflicts of Interest

The authors declare no conflicts of interest.

## Supporting information


**Data S1:** fsb270958‐sup‐0001‐supinfo.pdf.

**Table S1:** List of primers used in this study, including gene targets and primer sequences (5′–3′).

## Data Availability

The data that support the findings of this study are available from the corresponding author upon reasonable request.
