# Supplementary material for: Intracellular and Extracellular Vesicle miRNA Signatures in Human iPSC‐Derived Neural Stem Cells and Floor Plate Progenitors
Source: FASEB J. 2025 Aug 28;39(16):e70958. doi: 10.1096/fj.202501157R (PMC12392061; doi:10.1096/fj.202501157R)
Supplement: Supplementary file 1 — Data S1: fsb270958‐sup‐0001‐supinfo.pdf. [file FSB2-39-e70958-s002.pdf]

Figure S1

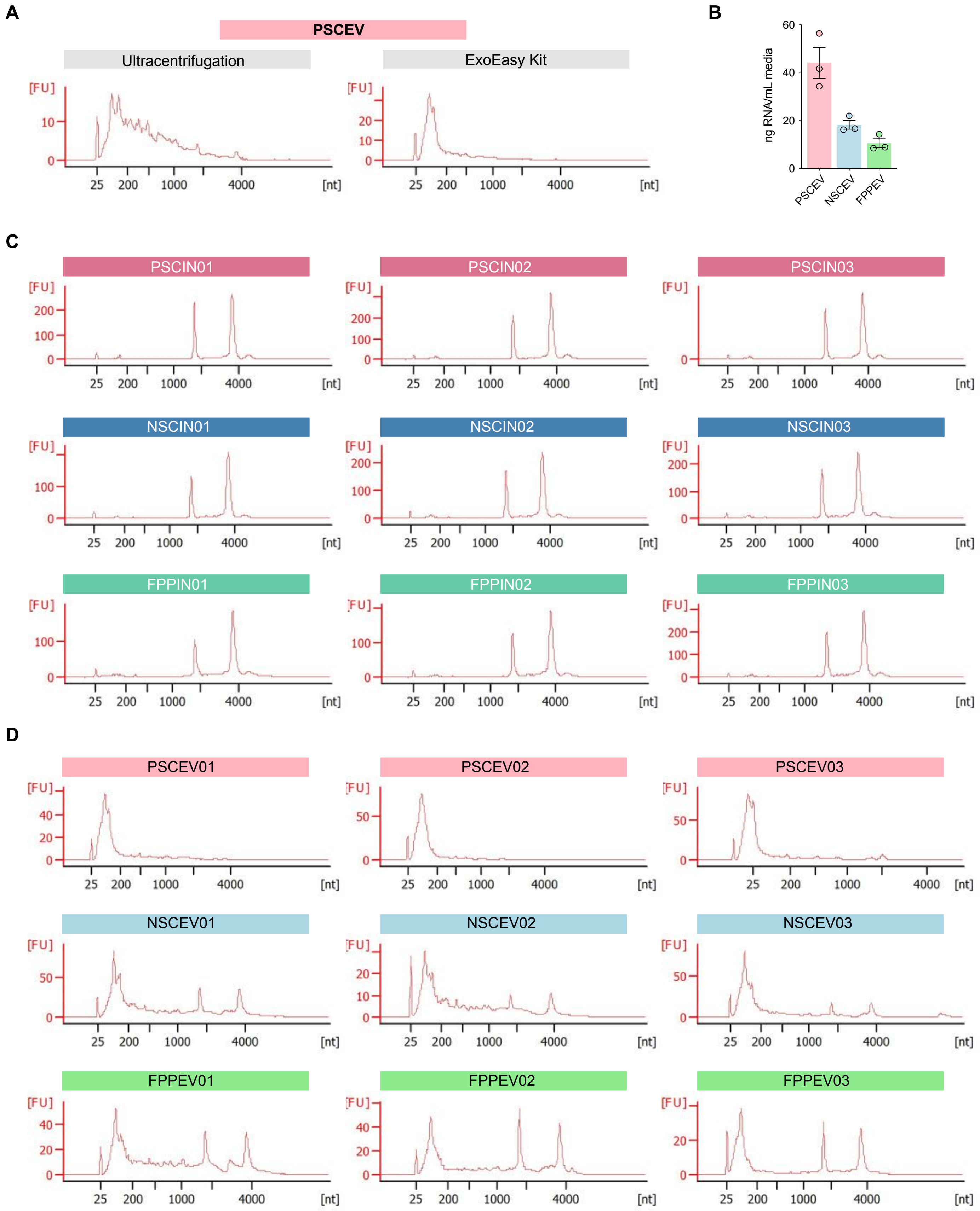

Supplemental Figure 1: Intracellular and EV RNA quality control by Bioanalyzer. **A.** Conditioned medium was collected from one pluripotent stem cells line (PSCEV01), processed by sequential spin steps (Fig.1C) and split in to two equal volume. Two EV isolation methods (Ultracentrifugation and ExoEasy Maxi kit) were tested simultaneously with equal volume of same medium source. EV RNA was extracted simultaneously and RNA quality was assessed by Bioanalyzer. **B**. EV RNA yield per mL of conditioned media. **C and D**. Bioanalyzer chromatograms of intracellular (**C**) and EV (**D**) RNA from all samples used in the study. Biological replicates are represented by the numbers (01, 02, 03). nt: nucleotides. FU: fluorescence units. Data are represented as mean +- SEM.

Figure S2

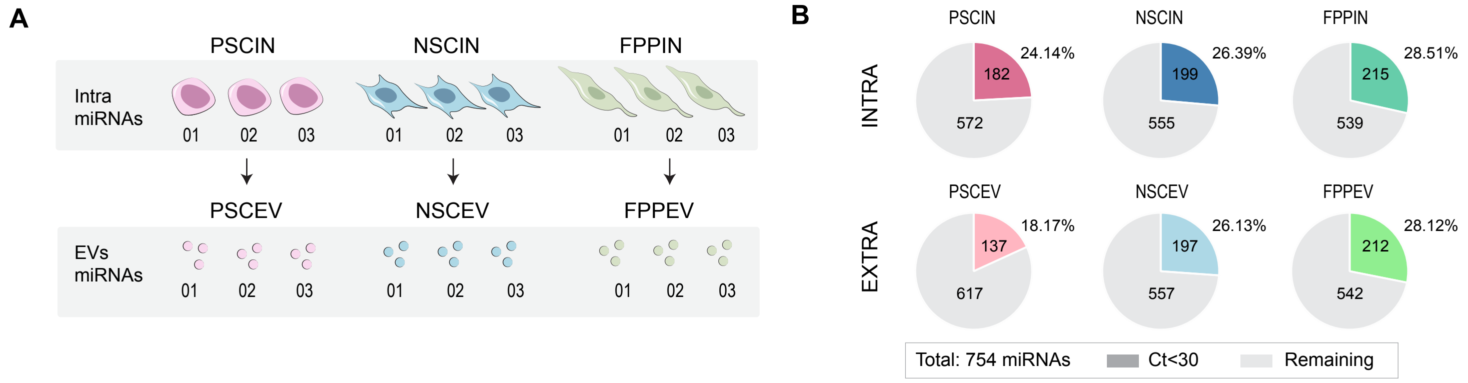

C

PSCIN	PSCEV	NSCIN	NSCEV	FPPIN	FPPEV
hsa-miR-302b-3p	hsa-miR-1274B	hsa-miR-17-5p	hsa-miR-1274B	hsa-miR-17-5p	hsa-miR-1274B
hsa-miR-367-3p	hsa-miR-1274A	hsa-miR-20a-5p	hsa-miR-20a-5p	hsa-miR-106a-5p	hsa-miR-20a-5p
hsa-miR-302d-3p	hsa-miR-302b-3p	hsa-miR-106a-5p	hsa-miR-17-5p	hsa-miR-20a-5p	hsa-miR-17-5p
hsa-miR-302a-3p	hsa-miR-720	hsa-miR-92a-3p	**hsa-miR-106a-5p**	hsa-miR-92a-3p	**hsa-miR-106a-5p**
hsa-miR-20a-5p	**hsa-miR-886-3p**	hsa-miR-720	**hsa-miR-1274A**	hsa-miR-720	**hsa-miR-1274A**
hsa-miR-17-5p	hsa-miR-302a-3p	hsa-miR-1274B	hsa-miR-92a-3p	hsa-miR-1274B	hsa-miR-92a-3p
hsa-miR-106a-5p	hsa-miR-302d-3p	hsa-miR-20b-5p	hsa-miR-720	hsa-miR-9-5p	hsa-miR-9-5p
hsa-miR-1274B	hsa-miR-20a-5p	**hsa-miR-1275**	hsa-miR-30c-5p	**hsa-miR-484**	hsa-miR-20b-5p
hsa-miR-302c-3p	hsa-miR-367-3p	hsa-miR-9-5p	hsa-miR-20b-5p	hsa-miR-218-5p	hsa-miR-720
hsa-miR-720	hsa-miR-17-5p	hsa-miR-484	hsa-miR-30b-5p	**hsa-miR-331-3p**	hsa-miR-191-5p
hsa-miR-92a-3p	hsa-miR-106a-5p	hsa-miR-30c-5p	hsa-miR-191-5p	hsa-miR-20b-5p	hsa-miR-30c-5p
hsa-miR-1274A	hsa-miR-302c-3p	**hsa-miR-320a**	hsa-miR-9-5p	**hsa-miR-1275**	hsa-miR-24-3p
**hsa-miR-1275**	hsa-miR-92a-3p	hsa-miR-30b-5p	**hsa-miR-130b-3p**	**hsa-miR-320a**	hsa-miR-218-5p
hsa-miR-20b-5p	**hsa-miR-886-5p**	**hsa-miR-331-3p**	**hsa-miR-130a-3p**	hsa-miR-24-3p	hsa-miR-30b-5p
**hsa-miR-302a-5p**	**hsa-miR-663b**	hsa-miR-191-5p	hsa-miR-93-5p	hsa-miR-30c-5p	**hsa-miR-221-3p**
hsa-miR-659-3p	**hsa-miR-30c-5p**	hsa-miR-93-5p	hsa-miR-106b-5p	hsa-miR-191-5p	**hsa-miR-335-5p**
**hsa-miR-221-3p**	hsa-miR-191-5p	hsa-miR-18a-5p	hsa-miR-18a-5p	hsa-miR-30b-5p	hsa-miR-106b-5p
**hsa-miR-18a-5p**	**hsa-miR-222-3p**	**hsa-miR-193b-3p**	**hsa-miR-219a-2-3p**	**hsa-miR-93-5p**	**hsa-miR-130b-3p**
hsa-miR-191-5p	hsa-miR-20b-5p	hsa-miR-130b-3p	hsa-miR-484	**hsa-miR-149-5p**	**hsa-miR-130a-3p**

Supplemental Figure 2: Assay characterization. A) Experimental design with nomenclature of groups. Intracellular (IN) miRNAs from pluripotent stem cell, neural stem cell and floor plate progenitors were named PSCIN, NSCIN and FPPIN, respectively. Accordingly, extracellular vesicles miRNAs groups were named PSCEV, NSCEV and FPPEV. hiPSCs from three individual (biological replicate: 01, 02 and 03) were used in the study and differentiated into NSCs and FPPs. EVs were isolated from each cell type and profile of IN and EVs miRNAs was compared among groups. **B)** Number and percentage of amplified miRNAs per group. **C)** Ranked top 20 most expressed miRNAs per group. It shows that the most expressed miRNAs in EVs reflect the most expressed correspondent intracellular miRNAs. The miRNAs which differ between each intra and EVs pair (PSC, NSC and FPP) are highlighted in bold.

Figure S3

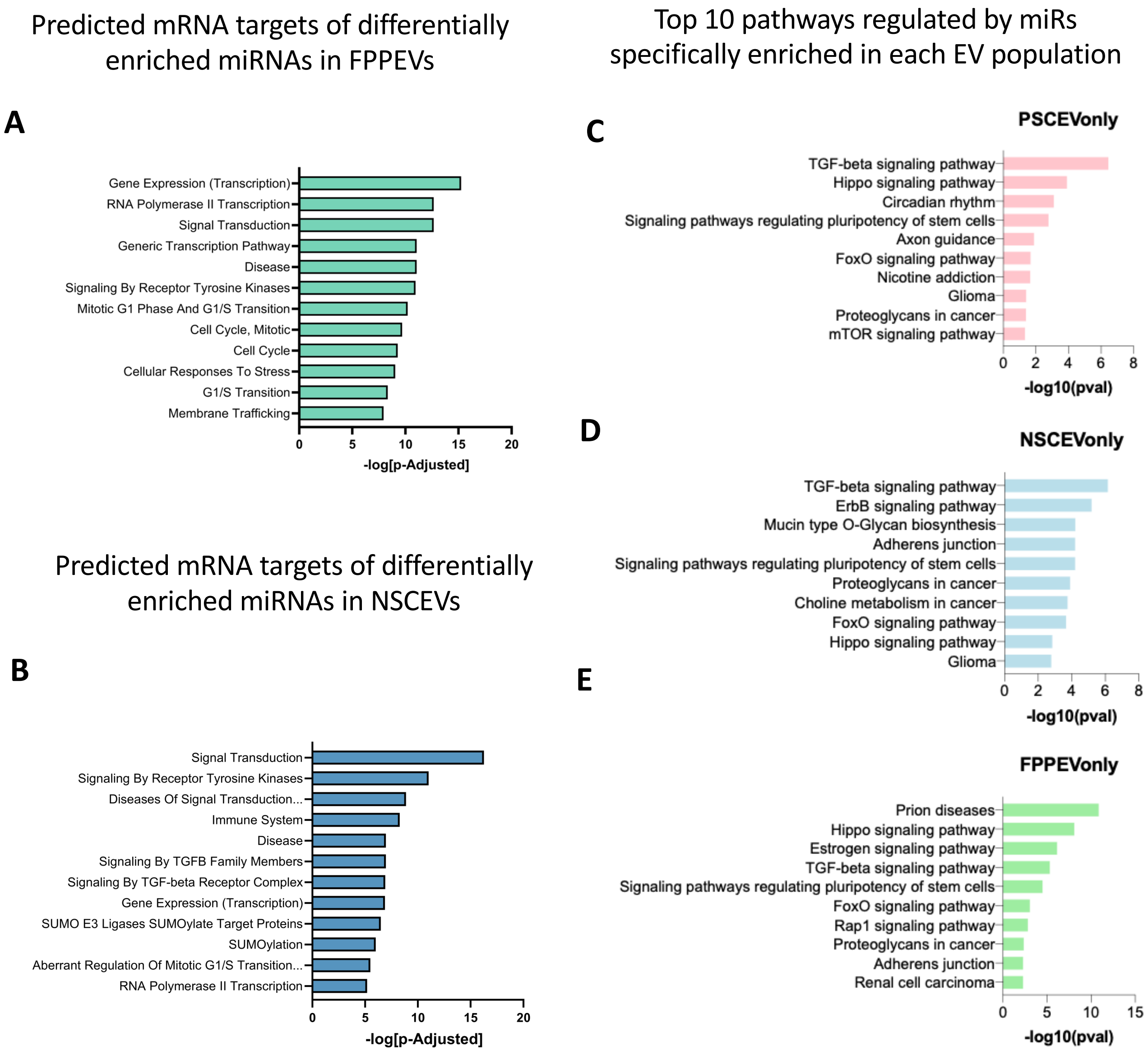

Supplemental Figure 3: Differential Pathway Enrichment of miRNAs in EVs Derived from PSCs, NSCs or FPPs. Predicted target pathway enrichment of 27 miRNAs differentially enriched in extracellular vesicles (EVs) derived from neural stem cells (NSCEVs) and floor-plate progenitors (FPPEVs). **A)** displays enriched pathways based on the 11 miRNAs upregulated in FPPEVs compared to NSCEVs. **B)** shows enrichment of pathways targeted by the 16 miRNAs upregulated in NSCEVs versus FPPEVs. **C-E)** Top 10 pathways predicted to be regulated by miRNAs specifically enriched in extracellular vesicles (EVs) derived from **(C)** pluripotent stem cells (PSCEVs), **(D)** neural stem cells (NSCEVs), and **(E)** floor-plate progenitors (FPPEVs), shown in separate panels. Pathway analysis was performed using over-representation analysis (ORA) of predicted targets of upregulated miRNAs in each condition.
