# Supplementary material for: Intracellular and Extracellular Vesicle miRNA Signatures in Human iPSC‐Derived Neural Stem Cells and Floor Plate Progenitors
Source: FASEB J. 2025 Aug 28;39(16):e70958. doi: 10.1096/fj.202501157R (PMC12392061; doi:10.1096/fj.202501157R)
Supplement: Supplementary file 2 — Table S1: List of primers used in this study, including gene targets and primer sequences (5′–3′). [file FSB2-39-e70958-s001.pdf]

Gene	Primer Name	Sequence (5' → 3')
CDC42	CDC42_F	GCCCGTGACCTGAAGGCTGTCA
CDC42	CDC42_R	TGCTTTTAGTATGATGCCGACACCA
RAC1	RAC1_F	CCTGATGCAGGCCATCAAG
RAC1	RAC1_R	AGTAGGGATATATTCTCCAGGAAATGC
WASL	WASL_F	GAACGAGTCCCTCTTCACTTTC
WASL	WASL_R	GTTCCGATCTGCTGCATATAACT
RAB10	RAB10_F	CAAGGGAGCATGGTATTAGGTTT
RAB10	RAB10_R	CTAACGTGAGGAACGCCTTTT
RAB13	RAB13_F	GATCCGCACTGTGGATATAGAGG
RAB13	RAB13_R	CCACGGTAGTAGGCAGTAGTTAT
RALGAPB	RALGAPB_F	GGAGATGGTGGCTAACTGGAG
RALGAPB	RALGAPB_R	GTAAAGCGTAGCAATCTGGAAGT
RALGAPA2	RALGAPA2_F	GCCCTAGTGTAGCTGATGTAAAG
RALGAPA2	RALGAPA2_R	TCTTCTCCCCTGATATGGCTG
GOLGB1	hGOLGB1_Fwd	GTGCTCAGGTCGTTGACTTG
GOLGB1	hGOLGB1_Rev	TGTTTCCACAGTGTTCCTCAAA
GOLGA2	hGOLGA2_Fwd	CACCCAAGGACAATGCTGCTA
GOLGA2	hGOLGA2_Rev	CCGCCATGCTAGTGAGACT
COG3	hCOG3_Fwd	CAGGAACCCTACATGAAGCCT
COG3	hCOG3_Rev	GGCCAGCATAGGTATAAATCCGT
USO1	hUSO1_Fwd	CCAGTGTGGGTTATTGCAGCA
USO1	hUSO1_Rev	GTTTACTTGGCAGCCTCGAAT
GORASP2	hGORASP2_Fwd	AGGTGGAATCAAATTCTCCTGC
GORASP2	hGORASP2_Rev	TCATGTGTTTCGATAAGGCTGAA
VPS37A	hVPS37A_Fwd	ACAGTAACCCAAGTGGGATGT
VPS37A	hVPS37A_Rev	AAGGACACCAAATGAAGGAGC
EXOC5	hEXOC5_Fwd	GTGCCAGGAGGGTGCTTATTT
EXOC5	hEXOC5_Rev	GCATCGGACTTCCTACATTCTTC
RAB8A	hRAB8A_Fwd	CGAAGGCCAACATCAATGTGG
RAB8A	hRAB8A_Rev	TCCGGTGTGATTTTGACTCCC
